# Perception and impact of university tutoring on academic performance: a case study at a Peruvian university

**DOI:** 10.3389/fpsyg.2026.1745043

**Published:** 2026-02-26

**Authors:** Jol M. Chirinos, Claudia Acra, Klinge Villalba

**Affiliations:** 1 Universidad de Málaga, Málaga, Spain; 2 Universidad Nacional Pedro Henriquez Urena, Santo Domingo, Dominican Republic; 3 Facultad de Ciencias de la Educación, Universidad Nacional de San Agustín de Arequipa, Arequipa, Peru

**Keywords:** academic performance, impact, perception, Peruvian university, university tutoring

## Abstract

University tutoring is a key strategy for inclusive higher education, aimed at supporting students’ academic performance and overall development. Its effectiveness, however, depends on tutor competencies, program structure, and students’ contextual conditions. This study examines students’ perceptions of tutoring and its relationship with satisfaction and academic performance at a public Peruvian university. A cross-sectional quantitative design was employed using survey data from 5,930 undergraduate students across 18 faculties during 2022. Descriptive analyses, multiple regression, and structural equation modeling (SEM) were conducted. Results show that students who participated in tutoring reported significantly higher satisfaction (*t* = 17.96, *p* < 0.001) and better academic performance. While the full regression model explained 40.3% of the variance, perceived tutoring impact alone accounted for approximately 8–9% in isolated models. Significant differences were observed across faculties, with students from biomedical fields reporting more favorable perceptions than those from social sciences and engineering. SEM analyses revealed that positive tutor perception is significantly associated with satisfaction, which in turn was associated with higher perceived academic impact. These relationships were moderated by students’ socioeconomic status and employment condition. Overall, findings indicate that while university tutoring is positively associated with student outcomes, its pedagogical effectiveness remains limited by implementation factors. The results highlight the need for context-sensitive, professionally trained, and structurally differentiated tutoring programs that account for students’ socioeconomic and labor conditions. This study provides large-scale empirical evidence from a Latin American context and offers practical insights for improving tutoring systems in higher education.

## 1 Introduction

University tutoring is an academic support process aimed at guiding and assisting students during one or more stages of their university education. It is carried out by a tutor whose purpose is to facilitate adaptation to the university environment, optimize academic performance, and promote the overall development of students at academic risk (González et al., 2024; Guffante et al., 2022). This support is based on the interaction between the tutor and the student, establishing a relationship rooted in trust, effective communication, and mutual commitment to achieving formative and personal goals (Elgegren, 2022; Pérez et al., 2024; Reséndiz and Zepeda, 2021).

Success in higher education has evolved; it is no longer limited to the acquisition of knowledge but extends to the development of transversal competencies and student well-being. In this context, university tutoring has become an essential pedagogical strategy to guide students in their academic and personal journey, especially those in adverse conditions or at academic risk (Esteban and Caro, 2023; Garay et al., 2024; Vita et al., 2021).

In the Latin American context and the Peruvian university system, the implementation of tutoring programs faces several challenges, ranging from students’ socioeconomic conditions to the management and resources available within universities. Often, these programs lack systematic evaluation of their impact and an analysis of the perceptions of key stakeholders: students, tutors, and administrators. This disconnect between design and practice limits the effectiveness of existing initiatives (García and Vilca, 2021; Navarro, 2025; Wakelin, 2021; Vita et al., 2021).

To address this gap and propose concrete guidelines for designing a contextualized and adaptable tutoring plan for the Peruvian reality, the present study analyzes the perception and impact of university tutoring. This work is based on the premise that a successful design must stem from a clear understanding of the needs and expectations of the university community (Alarcón et al., 2024; Chaverra et al., 2023; Martínez et al., 2022). The relevance of this study is related to higher academic performance for at-risk students in Peru and serve as a tool against university dropout by strengthening student support systems. Its objectives are to explore the correlations and perceptions of students regarding the tutoring service (Escalante et al., 2023).

University tutoring has become a key strategy in the holistic educational process of students, especially in the context of the massification of access to higher education and the diversity of student trajectories. This role has gained central importance, extending beyond mere academic support (Esteban and Caro, 2023; Cruzata et al., 2020; Pantoja et al., 2022). The tutor’s role has evolved from its traditional conception into a formative agent committed to the student’s development, acting as a critical companion, a facilitator of reflective processes, and a guide through the student’s academic journey (Cruzata et al., 2020).

Tutoring actions should be linked to the development of personal, academic, and professional competencies (Guffante et al., 2022; Moreno et al., 2023; Walker, 2020). It contributes to the student’s sense of belonging and encourages a critical attitude toward their education. It is emphasized that a non-directive educational relationship is necessary, where active listening and empathetic understanding are foundational to the tutoring relationship (Alarcón et al., 2024; Valdez et al., 2024; Yucra, 2022).

In the Latin American context, tutoring is recognized as an effective mechanism to reduce dropout rates, improve retention, and facilitate university adaptation (Benites, 2020; Escalante et al., 2023; Esquivel and Basilio, 2024). Tutoring enhances students’ responsibility and autonomy, which in turn boosts academic achievement and leads to significant improvements in soft skills such as leadership, communication, and decision-making. It plays an essential role in academic, institutional, and personal integration, with three complementary modalities: individual, group, and peer tutoring (Alcarraz and Sanchez, 2021; Benites, 2020; Bernardo et al., 2021).

The value of student perception depends on how students perceive and experience the quality of support, extending beyond the content provided. Emotional components should be explicitly included in tutoring programs, recognizing the importance of emotional sensitivity in addressing the psychosocial distress associated with the university transition (Angulo, 2021; Arámburo, 2023; Valdez et al., 2024). Successful programs share attention to individual needs, continuous teacher training, and an optimal relationship between the number of tutors and students (Alegre et al., 2024; Pari et al., 2024). Tutoring plans should consider the structural conditions of universities and the socio-emotional characteristics of students, integrating approaches to well-being and diversity (Elgegren, 2022; Vita et al., 2021).

The 21st-century university faces a dilemma between humanistic education and utilitarian orientation, with tutoring emerging as an alternative to personalize learning and facilitate adaptation to institutional and labor environments (Espinales et al., 2022; Gonzáles and Martínez, 2020; Martínez et al., 2020; Yana et al., 2024). Tutoring plays a role in minimizing constant failure rates and dropout, strengthening students’ capacities through individual or group guidance (Escalante et al., 2023). It must address students in an integrated manner, with clear objectives and continuous evaluation, as its effectiveness depends on the commitment and specialized training of the faculty (Medianero, 2017; Pantoja et al., 2022; Reséndiz and Zepeda, 2021).

Moreover, it is an effective mechanism for developing generic competencies such as critical thinking and self-regulation (Arámburo, 2023; Navarrete and Tomé, 2022; Yana et al., 2024). Student perception of tutoring is linked to the strengthening of interpersonal skills and the consolidation of transversal competencies. From an institutional perspective, it is necessary to document the importance of defining the scope of action, providing continuous training to tutors, and having monitoring systems to feed back the program based on evidence (Angulo, 2021; Arakaki et al., 2019; Guffante et al., 2022).

This study aims to provide large-scale empirical evidence on the perception and impact of university tutoring in a Latin American context, an understudied setting. Specifically, it seeks to: (1) quantify the relationship between tutoring, student satisfaction, and academic performance using quantitative analytical methods; (2) examine the moderating role of socioeconomic status and employment conditions through regression and structural equation modeling; and (3) identify critical gaps—particularly between students’ valuation of tutoring as a principle and their perception of its pedagogical execution—in order to inform the design of more effective and context-sensitive tutoring programs.

Rather than asking whether tutoring works, this study advances the literature by examining how tutoring is perceived, under which conditions it is more strongly associated with academic outcomes, and why students may simultaneously value tutoring while critically evaluating its implementation. This distinction is largely absent from prior research in Latin American higher education contexts.

## 2 Materials and methods

This study follows an observational, cross-sectional design. Tutoring participation was not randomly assigned; instead, it was institutionally mandated as part of the university’s academic support system. In accordance with policies established by the Academic Vice-Rectorate, tutoring is a compulsory component of undergraduate education. Faculty members are formally assigned tutoring responsibilities—including specific student groups, academic sections, or semesters—as part of their teaching workload, and these tutoring hours are financially remunerated.

Tutors are allocated to student groups by the Faculty or School of Psychology, which oversees the tutoring system and assigns students based on institutional criteria rather than student choice. Students identified as being at academic risk due to low academic performance or repeated course failure are systematically included in the tutoring program. Although assignment to tutoring is institutional rather than voluntary, levels of student engagement and interaction with tutors may vary, which precludes causal inference. Consequently, all comparisons between students who did and did not actively receive tutoring should be interpreted with caution and understood as associative rather than causal.

The main variables were defined as follows. Tutor perception and satisfaction with tutoring were treated as independent and mediating variables within the SEM framework. Perceived academic impact and grade point average were modeled as outcome variables. Socioeconomic status and employment condition were specified as moderators, while gender, age, scholarship status, and academic field were included as control variables.

The study adopted a quantitative approach with a descriptive level. To deepen the understanding of the relationships between the studied variables and provide a more comprehensive view of the impact of university tutoring on academic performance, the analysis was complemented with a Structural Equation Modeling (SEM) approach (Hair et al., 2022; Kline, 2023).

The analysis was conducted using the Maximum Likelihood (ML) method, suitable for large samples and multivariate normal distributions (Byrne and Cahyono, 2022). Before estimating the structural model, assumptions of multicollinearity, normality, absence of extreme outliers, and sample adequacy were checked using the KMO index (> 0.80) and Bartlett’s Test of Sphericity (*p* < 0.001), confirming the suitability of the data for prior confirmatory factor analysis (Schumacker and Whittaker, 2022).

The model was estimated based on two main components. First, the measurement model was used to examine the convergent and discriminant validity of the dimensions associated with university tutoring. Standardized factor loadings above 0.60 and composite reliability coefficients (CR > 0.70) demonstrated adequate internal consistency. Additionally, the Average Variance Extracted (AVE) exceeded the recommended threshold of 0.50, confirming satisfactory convergent validity.

Second, the structural model allowed the evaluation of the hypothesized relationships between latent and observed variables, estimating standardized regression coefficients (*β*), statistical significance (*p* < 0.05), and the proportion of explained variance (R^2^) for each dependent variable.

The global model fit evaluation was conducted through various indices, according to international consensus criteria. A relative chi-square (χ^2^/df) value lower than 3.00 was considered indicative of an acceptable fit. Furthermore, the Comparative Fit Index (CFI) and Tucker–Lewis Index (TLI) with values equal to or higher than the recommended thresholds reflected an adequate model fit. Finally, values of the Root Mean Square Error of Approximation (RMSEA) and Standardized Root Mean Square Residual (SRMR) equal to or lower than 0.08 indicated a satisfactory match between the theoretical model and empirical data (Byrne and Cahyono, 2022; Hair et al., 2022; Kline, 2023).

Although Bartlett’s Test of Sphericity yielded a relatively modest chi-square value (χ^2^ = 295.47, *p* < 0.001), this result should be interpreted in light of the reduced number of observed variables included in the factor analysis. In this context, the significance of the test confirms the presence of sufficient inter-item correlations to justify factor extraction, in combination with a high KMO index (0.88).

### 2.1 Participants

The target population consisted of undergraduate students from a public Peruvian university, belonging to 47 professional schools across 18 faculties, grouped into three major academic areas: Biomedical, Social Sciences, and Engineering.

The sample included 5,930 students selected through stratified probability sampling by faculty and professional school, ensuring institutional representativeness. Participants’ ages ranged from 18 to 25 years, with a mean age of 21.2 years (SD = 2.8). Inclusion criteria required that participants be of legal age, enrolled in the current academic cycle, and have voluntarily accepted informed consent before participating in the study.

The instrument was applied in a self-administered and anonymous manner through the university’s digital platforms, ensuring data confidentiality and compliance with ethical principles related to informed consent.

### 2.2 Instrument

The Student Satisfaction Survey toward Tutoring was used in this study. This instrument was developed and validated by the university’s Psychopedagogical Support Office as part of its institutional evaluation system, with the purpose of assessing students’ perceptions regarding the quality and effectiveness of tutoring support services.

The validation process focused on examining the internal structure and reliability of the instrument. Construct validity was assessed through Exploratory Factor Analysis (EFA). Prior to factor extraction, data suitability was confirmed using the Kaiser–Meyer–Olkin (KMO) measure of sampling adequacy, which reached a value of 0.88, and Bartlett’s Test of Sphericity, which was statistically significant (χ^2^ = 295.47, *p* < 0.001), indicating sufficient inter-item correlations for factor analysis.

The EFA identified three coherent latent factors corresponding to the core dimensions of the tutoring experience: socioeconomic, academic, and tutoring-related components. Together, these factors explained approximately 80% of the total variance, supporting a solid and theoretically consistent factorial structure. All retained items exhibited factor loadings above 0.70, indicating strong associations between observed variables and their respective latent constructs.

Although the chi-square value obtained in Bartlett’s Test was relatively modest, this result is attributable to the limited number of observed variables included in the analysis. In conjunction with the high KMO value, these findings confirm the adequacy of the data for factor analysis and support the psychometric soundness of the instrument for research purposes in the present context.

## 3 Results

### 3.1 Sample characteristics and preliminary analyses

The study included 5,930 undergraduate students from 18 faculties at a Peruvian university, with a mean age of 21.2 years (SD = 2.8). Participants were distributed across three major academic areas: Biomedical Sciences, Social Sciences, and Engineering. All participants were of legal age, were currently enrolled, and provided informed consent prior to participation.

### 3.2 Differences in tutoring satisfaction and impact by participation status and faculty

An independent-samples Student’s *t*-test revealed that students who received tutoring reported significantly higher satisfaction levels than those who did not participate (*t* = 17.96, *p* < 0.001), with a mean difference of 0.46 points (95% CI [0.41, 0.51]). This result was confirmed by a non-parametric Mann–Whitney U test (U = 1,796,025.0, *p* < 0.001). The magnitude of this difference corresponds to a moderate effect size (Cohen’s d ≈ 0.46), indicating that the observed difference is not only statistically significant but also practically meaningful.

Significant differences in perceived tutoring impact were observed across faculties (Table 1). A one-way ANOVA confirmed that these differences were statistically significant [*F*(17, 5,912) = 22.84, *p* < 0.001, η^2^ = 0.062], indicating that faculty affiliation accounted for approximately 6.2% of the variance in perceived tutoring impact. *Post hoc* Tukey tests showed that students from Biomedical faculties reported significantly higher perceived impact than those from Social Sciences (*p* < 0.01) and Engineering faculties (*p* < 0.001), while students from Social Sciences reported higher perceived impact than those from Engineering (*p* < 0.01).

**Table 1:** Perceived impact of tutoring by faculty.

Faculty	*n*	M	SD
Medicine	365	2.87	0.67
Nursing	360	2.83	0.68
Biological Sciences	314	2.81	0.70
Psychology, Industrial Relations, and Communication Sciences	350	2.73	0.70
Educational Sciences	350	2.77	0.71
Agronomy	387	2.72	0.69
Management	425	2.68	0.73
Historical-Social Sciences	292	2.66	0.75
Natural and Formal Sciences	271	2.63	0.72
Accounting and Financial Sciences	316	2.61	0.74
Philosophy and Humanities	190	2.60	0.74
Law	429	2.59	0.78
Production and Services	332	2.58	0.75
Economics	268	2.55	0.79
Architecture	255	2.49	0.76
Process Engineering	319	2.47	0.76
Geology, Geophysics, and Mining	265	2.44	0.77
Civil Engineering	442	2.41	0.80

ANOVA: *F*(17, 5,912) = 22.84, *p* < 0.001, η^2^ = 0.062. Post-hoc comparisons: Biomedical > Social Sciences (*p* < 0.01), Biomedical > Engineering (*p* < 0.001), Social Sciences > Engineering (*p* < 0.01).

### 3.3 Regression analysis: impact of tutoring and socioeconomic factors

A multiple regression analysis was conducted to examine predictors of academic performance (Table 2). The overall model was statistically significant [*F*(9, 5,930) = 445.8, *p* < 0.001] and explained 40.3% of the variance in academic performance (*R*^2^ = 0.4034). Perceived tutoring impact emerged as the strongest predictor (*β* = 0.616, *p* < 0.001).

**Table 2:** Multiple regression analysis: predictors of academic performance.

Predictor	Coef.	Std. error	*t*	*p*	Std. Beta
Tutoring impact	2.0401	0.0330	61.76	< 0.001	0.6163
Gender (Female)	0.2117	0.0477	4.44	< 0.01	0.0447
Age	0.0034	0.0087	0.39	0.700	0.0040
Employed (Yes = 1)	−0.7597	0.0547	−13.90	< 0.001	−0.1379
Scholarship (Yes = 1)	0.4649	0.0864	5.38	< 0.001	0.0543
Income (Medium vs. Low)	0.1510	0.0473	3.19	0.001	0.0317
Income (High vs. Low)	0.3725	0.0611	6.09	< 0.001	0.0671
Field (Biomedical vs. Engineering)	0.2482	0.0658	3.77	< 0.001	0.0498
Field (Social Sciences vs. Engineering)	−0.0792	0.0498	−1.59	0.112	−0.0158
Intercept	9.0065	0.2114	42.60	< 0.001	

*R*^2^ = 0.4034; Adjusted *R*^2^ = 0.4027; *F*(9, 5,920) = 445.8, *p* < 0.001.

Women reported slightly higher academic averages than men (*β* = 0.045, *p* < 0.01), whereas students who worked had lower academic scores (*β* = −0.138, *p* < 0.001). Scholarship receipt (*β* = 0.054, *p* < 0.001) and higher income levels (medium: *β* = 0.032, *p* = 0.001; high: *β* = 0.067, *p* < 0.001) were positively associated with academic performance. Students from biomedical faculties outperformed those from engineering faculties (*β* = 0.050, *p* < 0.001), while no significant difference was observed between social sciences and engineering students (*p* = 0.112).

### 3.4 Structural equation models

#### 3.4.1 Measurement model: tutor perception and satisfaction

The measurement model for tutor perception and satisfaction demonstrated adequate fit indices and satisfactory convergent validity (Table 3). All standardized factor loadings were statistically significant (*p* < 0.001) and exceeded the recommended threshold of 0.60, ranging from 0.67 to 0.88. Composite reliability coefficients were 0.91 for Tutor Perception and 0.89 for Satisfaction, while the Average Variance Extracted values were 0.63 and 0.59, respectively, confirming adequate internal consistency.

**Table 3:** Measurement model: standardized factor loadings for tutor perception and satisfaction.

Indicator	Latent variable	Standardized loading (*λ*)	SE	*z*	*p*
Tutor’s skills	Perception	0.81	0.03	27.00	< 0.001
Importance of the role	Perception	0.74	0.04	20.15	< 0.001
Empathic attitude	Perception	0.88	0.03	29.33	< 0.001
Perceived effectiveness	Satisfaction	0.67	0.05	13.40	< 0.001
Support received	Satisfaction	0.82	0.04	20.50	< 0.001
Academic usefulness	Satisfaction	0.84	0.03	22.61	< 0.001

CR (Perception) = 0.91; AVE = 0.63. CR (Satisfaction) = 0.89; AVE = 0.59.

#### 3.4.2 Structural path: tutor perception and satisfaction

The structural model examining the relationship between tutor perception and satisfaction demonstrated excellent fit indices (χ^2^/df = 2.97, CFI = 0.96, TLI = 0.94, RMSEA = 0.047, SRMR = 0.041). The path from Tutor Perception to Satisfaction was positive and statistically significant (*β* = 0.57, SE = 0.05, z = 11.40, *p* < 0.001), explaining 32% of the variance in student satisfaction (R^2^ = 0.32) (Table 4).

**Table 4:** Standardized structural coefficients (perception → satisfaction model).

Structural relationship	Unstandardized coefficient (B)	SE	*z*	*p*	Standardized coefficient (*β*)	R^2^
Satisfaction ← Perception	0.62	0.05	11.40	< 0.001	0.57	0.32

Model fit: *χ*^2^/gl = 2.97, CFI = 0.96, TLI = 0.94, RMSEA = 0.047, SRMR = 0.041.

#### 3.4.3 Structural path: satisfaction and perceived impact

The measurement model for satisfaction and perceived impact demonstrated adequate convergent validity, with standardized factor loadings ranging from 0.64 to 0.85 (all *p* < 0.001), composite reliability values of 0.89 and 0.88, and Average Variance Extracted (AVE) values of 0.61 and 0.59, respectively. The structural model showed satisfactory fit (χ^2^/df = 3.05, CFI = 0.95, TLI = 0.93, RMSEA = 0.049, SRMR = 0.045). Satisfaction with tutoring significantly predicted perceived impact on academic performance (*β* = 0.54, *p* < 0.001), explaining 29% of the variance in perceived impact (*R*^2^ = 0.29) (Table 5).

**Table 5:** Standardized structural coefficients (satisfaction → perceived impact model).

Structural relationship	Unstandardized coefficient (B)	SE	*z*	*p*	Standardized coefficient (*β*)	*R* ^2^
Perceived Impact ← Satisfaction	0.59	0.05	10.98	< 0.001	0.54	0.29

Model fit: *χ*^2^/gl = 3.05, CFI = 0.95, TLI = 0.93, RMSEA = 0.049, SRMR = 0.045.

#### 3.4.4 Moderating effects of socioeconomic status and employment

A multigroup SEM analysis was conducted to examine whether socioeconomic status and employment status moderated the relationship between satisfaction with tutoring and perceived impact on academic performance (Table 6). For socioeconomic status, partial moderation was observed (ΔCFI = 0.006), with the association of satisfaction on perceived impact increasing across low (*β* = 0.48), medium (*β* = 0.56), and high (*β* = 0.62) socioeconomic groups. For employment status, significant moderation was identified [ΔCFI = 0.012, Δχ^2^(1) = 11.45, *p* = 0.001], with a stronger association among unemployed students (*β* = 0.58) compared to employed students (*β* = 0.46).

**Table 6:** Moderating effects on the satisfaction → perceived impact path.

Moderator	Group	Standardized *β*	SE	*z*	*p*	*R* ^2^	Moderation test
Socioeconomic status	Low	0.48	0.06	8.13	< 0.001	0.26	ΔCFI = 0.006
	Medium	0.56	0.05	10.85	< 0.001	0.31	Partial moderation
	High	0.62	0.07	8.86	< 0.001	0.36	
Employment status	Employed	0.46	0.06	7.66	< 0.001	0.24	ΔCFI = 0.012*
	Unemployed	0.58	0.05	11.35	< 0.001	0.33	Significant moderation

Difference between groups: Δχ^2^(1) = 11.45, *p* = 0.001.

#### 3.4.5 Relationship between tutoring participation and service evaluation

A chi-square test of independence revealed a statistically significant association between receiving tutoring and the evaluation of service quality [χ^2^(3, *N* = 5,930] = 146.27, *p* < 0.001), with a small-to-moderate effect size (Cramér’s V = 0.157) (Table 7). Students who received tutoring were more likely to rate the service as “unsatisfactory” (51.9%) compared to those who did not receive tutoring (13.6%), whereas students who did not receive tutoring reported higher proportions of “good” (49.3%) and “very good” (15.6%) evaluations. This apparent contradiction suggests that satisfaction with tutoring outcomes differs from evaluations of service quality.

**Table 7:** Relationship between tutoring use and perceived quality.

Perceived quality	Received tutoring (*n* = 1,331)	Did not receive tutoring (*n* = 4,599)	Total
Very good	124 (9.3%)	716 (15.6%)	840 (14.2%)
Good	272 (20.4%)	2,269 (49.3%)	2,541 (42.9%)
Fair	244 (18.3%)	988 (21.5%)	1,232 (20.8%)
Unsatisfactory	691 (51.9%)	626 (13.6%)	1,317 (22.2%)
Total	1,331 (100%)	4,599 (100%)	5,930 (100%)

χ^2^(3, *N* = 5,930) = 146.27, *p* < 0.001, Cramér’s V = 0.157.

Students who did not receive tutoring evaluated the tutoring service based on institutional reputation, indirect information, or general expectations, rather than on direct personal experience with the tutoring process. Therefore, their ratings reflect perceived institutional quality rather than experienced tutoring quality. In contrast, students who participated in tutoring assessed the service based on direct pedagogical interaction, which likely resulted in more critical and experience-based evaluations.

A Mann–Whitney U test confirmed that the perceived quality of the tutoring service was significantly lower among students who received tutoring (Mdn = 4, “Unsatisfactory”) than among those who did not receive tutoring (Mdn = 3, “Fair”) (U = 2,731,600, *z* = −12.64, *p* < 0.001), with a small effect size (*r* = 0.16) (Table 8). This value corresponds to a small practical difference between groups, reinforcing that the observed discrepancy reflects perceptual rather than substantial differences in service quality evaluations.

**Table 8:** Comparison of perceived quality of tutoring service by tutoring participation.

Group	*N*	Mean	SD	Median	*U*	*z*	*p*	*r*
Received tutoring	1,331	3.08	0.71	4 (“Unsatisfactory”)	2,731,600	−12.64	< 0.001	0.16
Did not receive tutoring	4,599	2.74	0.82	3 (“Fair”)				

An important and conceptually relevant finding emerges from the comparison between tutoring participation and perceived service quality. Students who did not receive tutoring tended to rate the tutoring service more positively than those who actually used it. Rather than constituting a methodological inconsistency, this pattern reveals a critical analytical gap in the literature between institutional reputation-based evaluations and experience-based evaluations of student support services.

Students who have not interacted directly with tutoring programs are likely to assess their quality based on indirect information, general institutional narratives, or expectations regarding academic support. In contrast, students who participated in tutoring evaluate the service based on concrete pedagogical interactions and personal experience. Consequently, the lower ratings observed among tutoring participants do not reflect rejection of tutoring as an institutional principle, but rather a more informed and critical appraisal of its actual implementation.

## 4 Discussion

The findings of this study highlight an understudied dimension of university tutoring: the distinction between perceived institutional value and experienced pedagogical quality. While prior research has predominantly focused on overall satisfaction indicators or academic outcomes associated with tutoring programs, fewer studies have examined how direct exposure to tutoring reshapes student evaluations, often revealing discrepancies between normative expectations and actual pedagogical practice. This distinction is particularly relevant in institutional contexts where tutoring is formally established as a central academic support mechanism.

By documenting this divergence, the present study contributes to a more nuanced understanding of why tutoring programs may be simultaneously valued and criticized within the same university setting. The results suggest that satisfaction with the idea or principle of tutoring should not be conflated with satisfaction regarding its pedagogical delivery. Evaluations provided by students who actively participated in tutoring appear to reflect a more informed and critical assessment of tutor practices, whereas non-users may rely on indirect perceptions or institutional narratives. This finding underscores the importance of incorporating the perspectives of actual service users when assessing tutoring quality and effectiveness.

Overall, this study provides large-scale empirical evidence on the relationships between students’ perceptions of tutoring, satisfaction, and academic performance in a Peruvian university context. Using regression analysis and structural equation modeling, the results reveal a complex landscape in which positive associations with academic outcomes coexist with important limitations in program implementation. Given the cross-sectional and observational design of the study, all reported relationships must be interpreted as statistical associations rather than causal effects. Although the SEM results support theoretically coherent predictive pathways, the absence of random assignment or longitudinal data precludes definitive causal inference.

This research makes three primary contributions to the literature on university tutoring in Latin American contexts. First, it provides empirical evidence from a large and institutionally representative sample (*N* = 5,930) in a region that remains underrepresented in higher education research. Second, it demonstrates that associations between tutoring-related variables and academic outcomes are moderated by socioeconomic status and employment conditions, highlighting the relevance of contextual and structural factors. Third, it identifies a critical dissonance between students’ valuation of tutoring as an institutional support mechanism and their more critical evaluations of its pedagogical execution, particularly regarding tutors’ instructional competencies.

The regression analyses indicate a strong association between positive perceptions of tutoring and academic performance. However, this association is not uniform across academic contexts. Significant differences between faculties, with more favorable perceptions in Biomedical Sciences than in Engineering and Social Sciences, suggest that disciplinary cultures, curricular structures, and academic workloads may substantially shape how tutoring is experienced and evaluated. These findings align with previous research indicating that standardized, one-size-fits-all tutoring models may limit effectiveness in diverse academic environments and that discipline-sensitive approaches may enhance tutoring outcomes (Alcocer et al., 2022; Capa et al., 2020; Elgegren, 2022; Sánchez and Mares, 2025).

Students with stronger academic skills or higher intrinsic motivation may both perform better academically and evaluate institutional programs more positively. While the models control for several sociodemographic factors, residual confounding cannot be ruled out.

A central paradox emerging from the results is that students who received tutoring reported higher levels of satisfaction with their academic experience while simultaneously expressing more critical evaluations of tutoring quality. This pattern suggests that direct engagement with tutoring does not necessarily lead to more favorable perceptions but rather facilitates more realistic and discerning evaluations. Students appear to recognize the potential value of tutoring while also identifying shortcomings in its pedagogical implementation, particularly in instructional guidance. Similar discrepancies between affective support and pedagogical effectiveness have been reported in prior studies on university tutoring and mentoring relationships (Guffante et al., 2022; Moreno et al., 2023; Walker, 2020).

Further analysis reveals disparities in how different dimensions of tutoring are evaluated. Interpersonal attributes such as empathy, respectful treatment, and availability received relatively high ratings, suggesting that tutors generally demonstrate adequate relational skills. This finding is consistent with conceptualizations of tutoring as a fundamentally relational practice that fosters trust and institutional belonging (Ceniz et al., 2024; Navarrete and Tomé, 2022; Topping, 2017). In contrast, pedagogical competencies and the perceived importance attributed to the tutoring role were evaluated less favorably, pointing to potential gaps in pedagogical training and role clarity. These deficiencies may constrain the educational effectiveness of tutoring interventions and help explain why tutoring-related variables, although statistically significant, account for a limited proportion of variance in academic performance.

The persistence of associations between tutoring variables and academic performance after controlling for sociodemographic factors reinforces the relevance of tutoring while simultaneously underscoring its contextual dependence. Paid employment and scholarship status emerged as significant correlates of academic outcomes, indicating that tutoring operates within a broader ecosystem of economic, temporal, and institutional constraints. These findings are consistent with prior research emphasizing that academic support initiatives interact with students’ socioeconomic realities rather than functioning as isolated interventions (Esteban and Caro, 2023; Cruzata et al., 2020; Pantoja et al., 2022).

Structural equation modeling results provide further theoretical coherence to the proposed framework. Tutor perception was positively associated with satisfaction with tutoring, explaining a substantial proportion of its variance. Satisfaction, in turn, was significantly associated with perceived academic impact. In this sense, satisfaction can be understood as a key intervening construct linking perceptions of the tutor–student interaction with students’ perceived academic benefits. This interpretation is consistent with prior work highlighting the role of tutoring experiences in shaping student confidence, self-regulation, and perceived academic usefulness (Álvarez, 2020; Castro et al., 2022). These findings support conceptual models that frame tutoring not merely as an informational service but as a relational and motivational process that may strengthen persistence and engagement in higher education (Hair et al., 2022; Kim and Lundberg, 2015; Klug and Peralta, 2019; Solis and Cañarte, 2025).

The inclusion of socioeconomic status and employment condition as moderators allowed for a more differentiated understanding of tutoring effectiveness. Partial moderation by socioeconomic status suggests that the association between satisfaction and perceived academic impact is stronger among students from medium- and high-socioeconomic backgrounds, potentially due to greater access to complementary learning resources that facilitate the application of tutor guidance (Elgegren, 2022; Esquivel and Basilio, 2024). Employment status showed significant moderation, with weaker associations observed among working students. Time constraints, academic stress, and reduced availability for extracurricular support activities may limit working students’ capacity to fully benefit from tutoring, as suggested by previous studies on student workload and academic engagement (Coila et al., 2023; Garay et al., 2024).

Taken together, these findings indicate that while university tutoring is positively associated with key academic and experiential outcomes, its effectiveness depends critically on pedagogical quality, contextual adaptation, and institutional support. Improving tutor training, clarifying pedagogical role expectations, and designing flexible tutoring modalities that account for students’ socioeconomic and employment conditions may enhance the contribution of tutoring programs to academic success and student well-being.

### 4.1 Toward an improved tutoring program: a proposal based on findings

Based on these findings, we propose an evidence-based tutoring support program designed to address the identified gaps between student expectations and current implementation. An effective tutoring program requires a rigorous approach based on four essential pillars: conceptualization, operationalization, human development, and evaluation, which must be addressed comprehensively to ensure sustainable outcomes (Table 9).

**Table 9:** Proposed components of the University Tutoring Support Program.

Dimension	Components	Actions	Success indicators
1. Diagnosis and planning	Needs analysis by faculty Identification of priority problems (Alegre et al., 2024; Elgegren, 2022)	Surveys with students and tutors Participatory workshops with practitioners and faculty Development of action plans by faculty	90% of faculty updated Plans aligned with needs
2. Teacher training	Tutoring skills Virtual platforms training Intercultural Education (Álvarez, 2021; Pari et al., 2024; Pacheco et al., 2025)	Bimonthly training-University-level tutoring training Use of case platforms and expert guidance	80% of tutors certified Reduction of gaps due to lack of technical knowledge
3. Tutoring implementation	Individual/group sessions Cross-cutting topics Integral monitoring (Paredes et al., 2022; Vidal and García, 2024)	Flexible schedules (in- person/virtual) Thematic guides on democratic coexistence, mental health, study techniques Referral to support services (psychology, social services)	70% student attendance 60% of students report improvement
4. Technology and accessibility	Unified platform Tutor Student Communication (Castro et al., 2022; Chani and Díaz, 2022; Martínez et al., 2022)	Platform use Scheduling and virtual session access Alerts for students at risk Access to digital resources	100% of tutors use the platform 50% of students access online resources
5. Promotion of values	Participatory democracy Interculturality	Semester campaigns Intercultural fairs Focus on human rights	80% student participation Reduction in discriminatory behaviors
6. Monitoring and evaluation	Prevention of discrimination (Medina et al., 2025; Pérez et al., 2024) Continuous feedback (Angulo, 2021)	Quarterly faculty reports Satisfaction surveys Support committees	30% improvement in student satisfaction 100% of faculty updated
7. Support for vulnerable students	Early identification Personalized academic support (Sánchez, 2025; Sánchez and Mares, 2025)	Alert system for students at risk Tutorials for advanced students Social support (scholarships, dining halls)	50% of students at risk improve 100% of detected cases attended

The initial conceptualization requires an institutional diagnosis to identify specific student population needs. Successful programs should address concrete academic challenges rather than offering generic support. Once the conceptual framework is defined, implementation should focus on operational aspects including tutor recruitment, selection, and training—critical components that should equip tutors with pedagogical tools to guide autonomous learning rather than merely solving problems. (Castro and Lara, 2018; Gonzáles, 2019; Yusof et al., 2022)

Systematic evaluation is essential to demonstrate program value and justify invested resources. Evaluation should be both formative (to monitor progress and make adjustments) and summative (to measure global impact). Quantitative data combined with qualitative insights provide a comprehensive view of program effects on academic success and student experience.

These results emphasize the need to design differentiated university tutoring strategies tailored to student diversity. Specifically, we recommend implementing flexible modalities (virtual or asynchronous) for working students and providing socioeconomic support programs that compensate for material resource gaps, which would promote equity in academic outcomes and optimize tutoring system effectiveness (Esteban and Caro, 2023; Espinales et al., 2022; Roy and Swargiary, 2024).

## 5 Limitations and future research directions

An important limitation is the potential influence of unobserved confounding variables, such as prior motivation, academic self-efficacy, or institutional engagement, which may simultaneously affect academic performance and positive perceptions of tutoring. As a result, the estimated associations should be interpreted as upper-bound estimates rather than causal effects.

A conceptual limitation of this study concerns the use of the term *tutoring*. Although the activities analyzed may resemble what is referred to as *mentoring* in other educational systems, in the Peruvian higher education context mentoring is neither formally defined nor institutionally regulated. Consequently, the study adopts the term *tutoring* as used in local policy and practice, with its operational definition explicitly specified in the Methods section. Caution is therefore warranted when generalizing these findings to contexts where tutoring and mentoring represent distinct and formally differentiated models.

Future research should employ longitudinal designs to better understand the temporal dynamics of tutoring effects. Experimental studies with random assignment to tutoring conditions would help establish causal relationships. Mixed-methods approaches could provide deeper insights into the qualitative experiences of both tutors and students. Additionally, research comparing different tutoring models across diverse institutional contexts would help identify best practices adaptable to various educational settings.

## 6 Conclusion

In light of the findings, university tutoring emerges as a dual-nature intervention in the Peruvian context studied: it demonstrates potential associations with improved academic performance and enhanced student trajectories, but its current implementation appears constrained by pedagogical competency gaps. The study reveals that students recognize the value of tutoring while remaining cognizant of its deficiencies. A positive perception of the tutor is associated with higher satisfaction with tutoring, and this satisfaction is linked to greater perceived impact on academic performance—relationships that are moderated by students’ socioeconomic and employment conditions.

The evidence suggests that merely having a tutoring program is insufficient; how it is designed and for whom it is adapted are crucial considerations. The marked differences between faculties are not mere variations but potential indicators of an overly standardized model within a diverse academic ecosystem. Twenty-first century tutoring cannot be merely an administrative protocol; it should be a reflective practice, contextualized to disciplinary needs, and supported by tutors who are not only companions but active facilitators of the learning process.

The future of tutoring lies not in its mere existence but in its capacity to evolve and adapt to student needs: it should transition from well-intentioned support to an intelligent strategy, integrated into the curriculum, and backed by substantial tutor training. Institutional commitment to ongoing tutor development, contextualized program design, and systematic evaluation will be essential to realize the full potential of university tutoring as a tool for educational enhancement and student support.

## Data Availability

The original contributions presented in the study are included in the article/supplementary material, further inquiries can be directed to the corresponding author.

## References

[ref1] Alarcón M. Vallejo M. Huerta M. Bahajin S. (2024). University tutoring-mentoring in emotional intelligence. Rev. Cienc. Salud Desarr. Hum. 5, 1162–1184. doi: 10.61368/r.s.d.h.v5i2.221

[ref2] Alcarraz L. Sanchez E. (2021). Tutorial action plan and its contribution to a university study program. Rev. Elec. Cal. En la Ed. Sup. 12, 1–6. doi: 10.22458/caes.v12i2.2857

[ref3] Alcocer F. Quijano R. Chuc G. (2022). Impact of tutor and tutoree gender in the higher education tutoring program in southeastern Mexico. Rev. Cienc. Salud Desarr. Hum. Rev. Electr. Tecnol. Educ. Soc. 9:18. https://www.ctes.org.mx/index.php/ctes/article/view/782

[ref4] Alegre A. Guevara D. Gerbi A. (2024). Psychometric analysis of the university tutoring service evaluation scale in first-year students in Lima, Peru. Rev. Gest. Pers. Tecnol. 17, 16–36. doi: 10.35588/z9e02n41

[ref5] Álvarez P. (2020). Practical guide: Questions and answers on how to develop orientation and tutorial action plans (POAT) in university teaching. San Cristóbal de La Laguna: University of La Laguna.

[ref6] Álvarez R. (2021). Virtual tutoring program for university students in the course on comprehension and text writing. Rev. Sci. 6, 231–247. doi: 10.29394/scientific.issn.2542-2987.2021.6.22.12.231-247

[ref7] Angulo A. (2021). Indicators of comprehensive tutoring from the perception of students in public universities in Mexico. Dilemas Contemp. 8:2512. doi: 10.46377/dilemas.v8i2.2512

[ref9001] Arakaki M. Dammert M. Mendoza N. Herrera D. (2019). Tutorís universitaria: aprendizajes y reflexiones a partir del programa de tutoría de la Facultad de Psicología en la Pontifica Universidad Católica del Perú. Blanco y Negro. 10, 12–23. https://revistas.pucp.edu.pe/index.php/enblancoynegro/article/view/21575/21204

[ref8] Arámburo J. (2023). Proposal for an instrument to evaluate the basic emotions of students within a university tutoring program. RIDE Rev. Iberoam. Investig. Desarr. Educ. 14:27. doi: 10.23913/ride.v14i27.1636

[ref10] Benites R. (2020). The role of academic tutoring in improving academic performance in university students. Conrado 16, 315–321. https://conrado.ucf.edu.cu/index.php/conrado/article/view/1602

[ref11] Bernardo C. Rivera N. Montes A. Mancilla B. (2021). Tutoring and training of university students. Form. Doc. 4, 14–31. doi: 10.31876/ie.v4i3.67

[ref12] Byrne B. Cahyono S. (2022). Structural equation modeling with AMOS. Taylor and Francis Group. Available online at: https://www.researchgate.net/publication/361909378_Structural_Equation_Modeling_With_AMOS (Accessed October 30, 2025).

[ref13] Capa L. Rojas W. Barreto L. (2020). Tutoring: procedure to determine the conditions for orientation and academic reinforcement. Conrado 16, 54–63.

[ref14] Castro J. Lara J. (2018). Academic tutoring in higher education: a strategy to face the challenges of accounting, administration, and computer science careers. EDUCIENCIA 2, 27–31. doi: 10.29059/educiencia.v2i1.35

[ref15] Castro L. Núñez L. García C. Tapia E. León C. (2022). Validity of the trust scale in affective tutoring and the usefulness of academic tutoring. Diálogos sobre Educ. Temas Act. Investig. Educ 13:24. doi: 10.32870/dse.v0i24.1080

[ref16] Ceniz L. Péez J. Rabí Á. (2024). The guiding competence of the university tutor in the academic and personal development areas. Didasc@Lia Didáct. y Educ 15, 299–317. https://dialnet.unirioja.es/servlet/articulo?codigo=9632820

[ref17] Chani M. A. Díaz J. (2022). Perception of comprehensive tutoring in students of a private university, Lima 2022. IGOBERNANZA 5, 119–132. doi: 10.47865/igob.vol5.n20.2022.225

[ref18] Chaverra D. Bolívar W. Calle G. (2023). Design of digital educational content to tutor academic writing. Educ. y Ciudad. 45:2821. doi: 10.36737/01230425.n45.2023.2821

[ref19] Coila A. Condori W. Ruelas R. Suaña M. Condori J. (2023). “University tutoring and academic performance in students of the language, literature, psychology, and philosophy program” in Desafíos y perspectivas de la educación II. ed. PACIFIC IDICAP (Puno: EIP), 187–200.

[ref20] Cruzata A. Del Valle J. Rodríguez I. Villareal C. (2020). University tutoring: Comprehensive support. 1st Edn. Lima: Fondo Editorial USIL.

[ref21] Elgegren J. (2022). University tutoring in the professional formative process. Educa UMCH 20, 218–233. https://dialnet.unirioja.es/descarga/articulo/9049212.pdf

[ref22] Escalante J. Medina C. Vásquez A. (2023). University dropout: an unresolved problem in Peru. Hacedor AIAPÆC 7, 60–72. doi: 10.26495/rch.v7i1.2421

[ref23] Espinales M. Durán A. Donoso M. (2022). The importance of the tutoring system within the formative process at the Universidad Técnica de Manabí (UTM), and its impact on institutional evaluation processes. Latam Rev. Latinoam. Cienc. Soc. Humanid. 3, 995–1010. doi: 10.56712/latam.v3i2.162

[ref24] Esquivel T. Basilio E. (2024). University tutoring: impact of its application on professional and personal development. Int. Educ. Technol. Rev. 9, 9–16. doi: 10.62701/revedutech.v9.5426

[ref9003] Esteban F. Caro M. (2023). The Cultivation of Critical Thinking through University Tutoring: A New Opportunity after Covid-19. Rev. Esp. Pedagog. 81, 73–90. https://www.jstor.org/stable/48713840

[ref25] Garay L. Martínez C. Michel E. Venegas B. Vera F. (2024). Impact of tutoring as a course on the adaptation and academic performance of new university students. Latam Rev. Latinoam. Cienc. Soc. Humanid. 5, 1992–2011. doi: 10.56712/latam.v5i6.3138

[ref26] García E. Vilca A. (2021). Tutoring action and academic performance of students in accounting, administrative sciences, and economics at the Universidad Nacional del Altiplano. Comuni@cción 12, 142–154. doi: 10.33595/2226-1478.12.2.516

[ref27] Gonzáles K. (2019). UNE tutoring model. Páginas SAC. Available online at: https://www.une.edu.pe/tecnologia/wp-content/uploads/2021/05/Modelo-de-tutoria-UNE.pdf (Accessed October 28, 2025).

[ref28] Gonzáles N. Martínez P. (2020). Relevance of transversal competencies in the professional development of graduates: student perception. Profesorado Rev. Currículum Form. Prof. 24, 388–413. doi: 10.30827/profesorado.v24i2.15041

[ref29] González N. González C. Martínez P. Pérez J. (2024). Flipped tutoring in higher education: student satisfaction with an experience of educational innovation. Rev. Electrón. Educare 28, 1–20. doi: 10.15359/ree.28-1.17280

[ref30] Guffante F. Guffante T. Barragán V. Meneses M. (2022). Impact of tutoring on the integral formation of university students. Podium Rev. Cienc. Tecnol. Cult. Fís. 17, 622–640. http://scielo.sld.cu/scielo.php?pid=S1996-24522022000200622&script=sci_abstract&tlng=es

[ref31] Hair J. Babin B. Anderson R. Black W. (2022). Multivariate data analysis. 8th Edn. Boston, MA: Cengage Learning.

[ref32] Kim Y. Lundberg C. (2015). A structural model of the relationship between student–faculty interaction and cognitive skills development among college students. Res. High. Educ. 57, 288–309. doi: 10.1007/s11162-015-9387-6

[ref33] Kline R. (2023). Principles and practice of structural equation modeling. 5th Edn. New York, NY: The Guilford Press.

[ref34] Klug M. Peralta N. (2019). University tutoring: perceptions of students and tutors about its use and functioning. Educare 23, 319–341. doi: 10.15359/ree.23-1.16

[ref35] Martínez P. Pérez F. González C. (2022). The tutor competencies of university professors: validation of a tool. Rev. Electr. Investig. Educ. 24, 1–15. doi: 10.24320/redie.2022.24.e03.4028

[ref36] Martínez P. Pérez J. González N. González C. Martínez M. (2020). University tutoring as seen by its students: proposals for improvement. Rev. Educ. Super. 49, 55–72. doi: 10.36857/resu.2020.195.1251

[ref9002] Medianero Y. (2017). La tutoría en la enseñanza universitaria: Una revisión sistemática de la literatura. Revista Semestral Acción y Reflexión Educativa. 42, 32–63. https://revistas.up.ac.pa/index.php/accion_reflexion_educativa/article/view/83/75

[ref37] Medina A. Amador C. Flores A. (2025). Peer tutoring program as a strategy to reduce failure and dropout in higher education institutions. Rev. Iberoam. Investig. Desarr. Educ. 15:30. doi: 10.23913/ride.v15i30.2332

[ref38] Moreno M. Cruz T. Muñoz I. (2023). The new tutoring in higher education within hybrid models. Rev. Educ. Desarr. 64, 17–26. https://www.cucs.udg.mx/revistas/edu_desarrollo/anteriores/64/RED_64_Completa.pdf

[ref39] Navarrete Z. Tomé J. (2022). University tutoring: a historical approach. Rev. Hist. Educ. Latinoam. 24, 209–230. doi: 10.19053/01227238.13989

[ref40] Navarro B. (2025). Challenges of academic tutoring in the Chilean university context. Rev. Peruana Educ. 7, 19–30. doi: 10.37260/repe.v7n13.9

[ref41] Pacheco M. Holguin K. Laurián D. Lee G. Laurián C. (2025). Influence of the tutoring program and academic performance from the perspective of university students. Rev. Investig. Acad. Sin Frontera 1:43. doi: 10.46589/riasf.v1i43.780

[ref42] Pantoja A. Colmenero M. Molero D. (2022). Conditioning aspects of university tutoring: a comparative study. Rev. Investig. Educ. 40, 33–49. doi: 10.6018/rie.373741

[ref43] Paredes D. Olórtegui R. Kaqui M. Camones E. (2022). Tutoring and counseling program in the academic performance of students in a professional education school. SCIÉNDO 25, 41–47. doi: 10.17268/sciendo.2022.005

[ref44] Pari M. Díaz Y. Mamani H. Valero V. (2024). Tutoring and academic performance in university students from the Peruvian Altiplano. RGSA 18, 1–17. doi: 10.24857/rgsa.v18n8-112

[ref45] Pérez F. González N. González C. Martínez P. (2024). University students’ evaluation of tutoring. Rev. Colomb. Educ. 91, 99–120. doi: 10.17227/rce.num91-16615

[ref46] Reséndiz M. Zepeda R. (2021). Framework for the function of university tutoring to support student retention with a cyber-systems transdisciplinary approach. RIDE. Rev. Iberoam. Investig. Desarr. Educ. 12:23. doi: 10.23913/ride.v12i23.1009

[ref47] Roy K. Swargiary K. (2024). Effectiveness of peer teaching in enhancing academic performance, engagement, and social-emotional outcomes. ResearchGate. Available online at: https://www.researchgate.net/publication/381540425_Effectiveness_of_Peer_Teaching_in_Enhancing_Academic_Performance_Engagement_and_Social-Emotional_Outcomes_among_High_School_Students_A_Quasi-Experimental_Study (Accessed September 24, 2025).

[ref48] Sánchez D. (2025). Tutoring as a strategy for inclusive education. Con-Ciencia Bol. Cienc. Esc. Preparatoria 12, 85–89. doi: 10.29057/prepa3.v12i23.14057

[ref49] Sánchez M. Mares A. (2025). Peer tutoring strategies for university students at academic risk. Espacio I+D, Innov. Más Desarr. 14:40. https://www.espacioimasd.unach.mx/index.php/Inicio/article/view/450

[ref50] Schumacker R. Whittaker T. (2022). A beginner’s guide to structural equation modeling. 5th Edn. Mahwah, New Jersey: Routledge.

[ref51] Solis Y. Cañarte L. (2025). Evaluation of student satisfaction in specialized tutoring for the business administration career: a multidimensional analysis. Ciencia Lat. Rev. Cienc. Multidiscip. 9, 11774–11797. doi: 10.37811/cl:rcm.v9i1.16753

[ref52] Topping K. (2017). Peer assessment: learning by judging and discussing the work of other learners. Interdiscip. Educ. Psychol. 1:7. doi: 10.31532/interdiscipeducpsychol.1.1.007

[ref53] Valdez S. Díaz A. Sotomayor R. Delgado R. (2024). Tutoring and teaching in undergraduate university programs. Tierra Nuestra 18, 107–127. doi: 10.21704/rtn.v18i1.1900

[ref54] Vidal L. García J. T. (2024). Institutional tutoring program from the student’s perspective in a higher education program for technical superior university studies. Ciencia Lat. Rev. Cienc. Multidiscip. 8, 1679–1701. doi: 10.37811/cl:rcm.v8i1.9563

[ref55] Vita A. Daura F. Montserrat M. (2021). University tutoring between Latin America and Europe: the case of the Universidad Austral (Argentina) and the University of Palermo (Italy). Rev. Panam. Pedagog. 31:2123. doi: 10.21555/rpp.v0i31.2123

[ref56] Wakelin E. (2021). Personal tutoring in higher education: an action research project on how to improve personal tutoring for both staff and students. Educ. Action Res. 31, 998–1013. doi: 10.1080/09650792.2021.2013912

[ref57] Walker B. (2020). Tackling the personal tutoring conundrum: a qualitative study on the impact of developmental support for tutors. Act. Learn. High. Educ. 23, 65–77. doi: 10.1177/1469787420926007

[ref58] Yana M. Coila A. Vargas D. Hancco D. Yana N. Adco H. (2024). Tutoring and academic performance in university students. Horiz. Rev. Investig. Cienc. Educ. 8, 80–92. doi: 10.33996/revistahorizontes.v8i32.706

[ref59] Yucra Y. (2022). University tutoring in pandemic times, a priority for students from the highland region of Puno. Rev. Hist. Educ. Latinoam. 23:37. doi: 10.19053/01227238.12705

[ref60] Yusof K. Shah N. Rauf A. (2022). Fostering academic performance of private university students through mentoring program. Eur. Proc. Multidiscip. Sci. 3, 438–452. doi: 10.15405/epms.2022.10.43

